# The normal D-dimer paradox: pulmonary artery intimal sarcoma masquerading as pulmonary embolism: a case report

**DOI:** 10.1093/ehjcr/ytag750

**Published:** 2026-10-10

**Authors:** Maicol Cortez-Sandoval, Milagros Palomino, Dayana Hernandez, Jorge M Aldea Saldaña, Oscar Rodríguez-Fernández

**Affiliations:** School of Human Medicine, Universidad Científica del Sur, Avenida Edgardo Rebagliati 490, Jesús María, Lima 15072, Peru; Department of Cardiology, Edgardo Rebagliati Martins National Hospital, Av. Edgardo Rebagliati 490, Jesús María, Lima 15072, Peru; Faculty of Medicine, National University of Cajamarca, Av. Atahualpa 1050, Cajamarca 06003, Peru; Faculty of Health Sciences, Peruvian University of Applied Sciences, Av. Prolongacón Primavera 2390, Santiago de Surco, Lima 15023, Peru; Department of Cardiology, Edgardo Rebagliati Martins National Hospital, Av. Edgardo Rebagliati 490, Jesús María, Lima 15072, Peru

**Keywords:** Right-Sided Heart Failure, Sarcoma, Pulmonary Artery, D-dimer, Vascular Neoplasms, Case report

## Abstract

**Background:**

Pulmonary artery intimal sarcoma is an exceptionally rare and aggressive malignancy that can closely mimic pulmonary embolism. Overlapping clinical and imaging features frequently lead to diagnostic anchoring and delayed recognition, with devastating prognostic consequences.

**Case summary:**

A 50-year-old woman presented with a nine-month history of progressive dyspnoea and cough and was admitted with hypotension, jugular venous distension, peripheral oedema, and systolic murmurs. Laboratory testing revealed a D-dimer level of 0.5 μg/mL (reference range 0.1–0.6 μg/mL). Transthoracic echocardiography showed right ventricular dilatation and a mass involving the pulmonary valve and main pulmonary artery. Computed tomography pulmonary angiography demonstrated an expansile lesion of the main pulmonary artery extending into both branches, with extraluminal involvement and new pulmonary nodules. Despite therapeutic anticoagulation for presumed pulmonary embolism, there was no clinical or radiological improvement. Histological diagnosis was obtained through biopsy of a breast lesion, revealing a high-grade sarcoma with vascular differentiation consistent with pulmonary artery intimal sarcoma. Given extensive local invasion and poor functional status (ECOG 4), curative treatment was not feasible, and the patient died 30 days after definitive diagnosis.

**Discussion:**

In the present case, pulmonary artery intimal sarcoma should be considered in patients with an atypical or refractory pulmonary embolism phenotype, particularly when a normal D-dimer level accompanies progressive pulmonary artery obstruction. Diagnostic clues include lack of response to anticoagulation, an expansile rather than occlusive intraluminal lesion, and extraluminal extension. Early recognition allows timely referral to specialized centres, which is critical given the poor prognosis once advanced local invasion has developed.

Learning points

- Pulmonary artery intimal sarcoma may closely mimic pulmonary embolism and should be considered in atypical or refractory cases.
- A normal D-dimer level and lack of response to anticoagulation should raise suspicion of a non-thrombotic intravascular process.
- Extrathoracic metastases may provide a safer diagnostic route when intrathoracic biopsy is high risk.

## Introduction

Pulmonary artery intimal sarcoma is an exceptionally rare and aggressive malignant tumour, with ∼400 cases having been reported in the literature to date, most described as isolated case reports.^1–4^ It arises from the intimal layer of the pulmonary artery and typically presents as an intraluminal mass that may extend longitudinally along the arterial wall, occasionally involving the pulmonary valve and, in advanced stages, the right ventricle.^1^

Although it can occur at any age, pulmonary artery intimal sarcoma is most frequently diagnosed in middle-aged adults. Prognosis remains poor, largely due to delayed diagnosis, with a reported median survival of ∼1–2 months in untreated patients. Survival may be prolonged in selected patients undergoing complete surgical resection, underscoring the importance of early recognition.^5^

Diagnosis is particularly challenging because both clinical presentation and initial imaging findings often mimic pulmonary embolism. Computed tomography pulmonary angiography is usually the first diagnostic modality, while cardiac magnetic resonance imaging and positron emission tomography–computed tomography may assist in differentiation by demonstrating contrast enhancement or metabolic activity within the intraluminal mass, features that favour a malignant vascular process over thromboembolic disease.^6^ We report a case initially managed as pulmonary embolism, in which rapid clinical deterioration and lack of response to anticoagulation ultimately led to the diagnosis of pulmonary artery intimal sarcoma, highlighting a critical diagnostic pitfall.

## Summary figure

**Figure:**
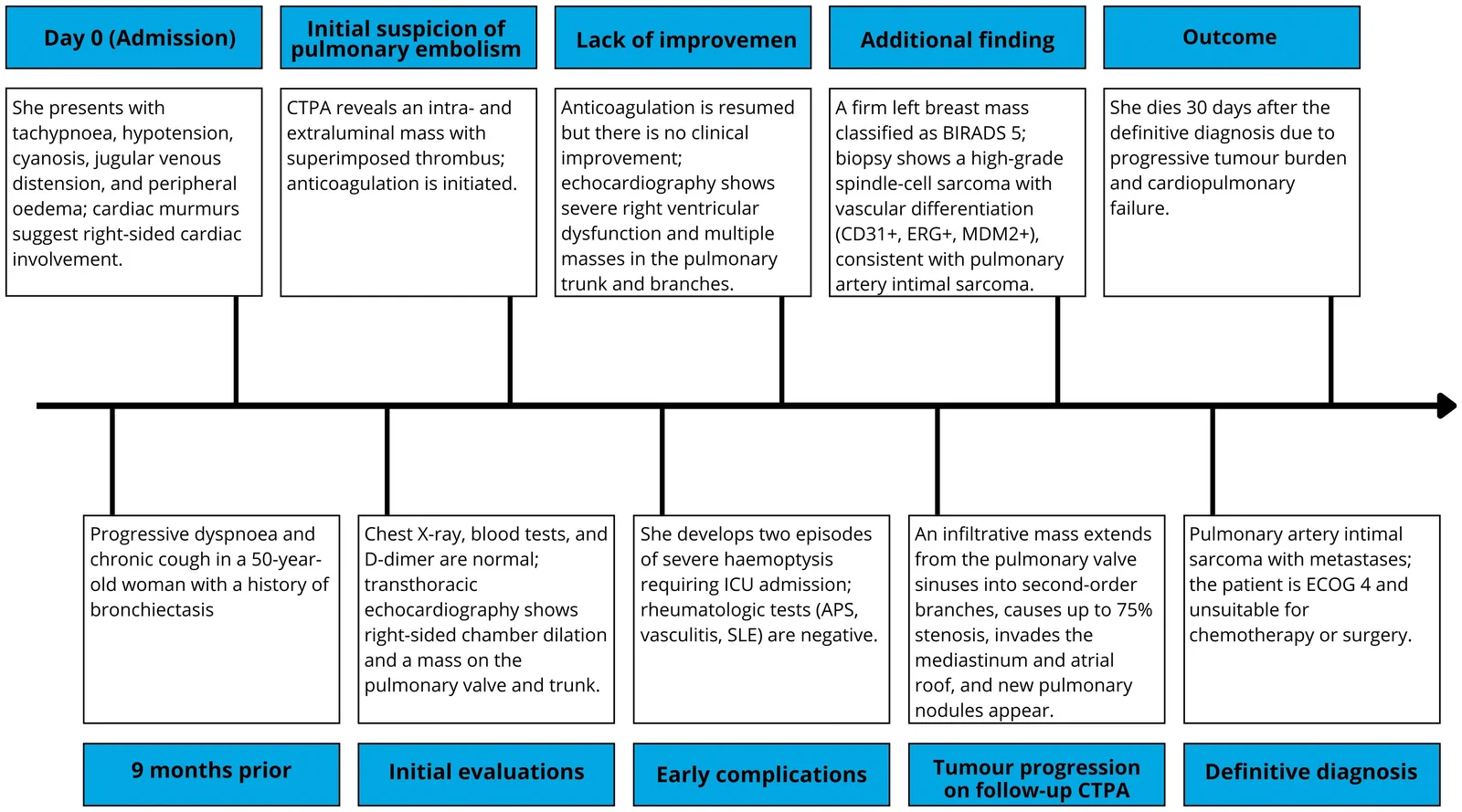


## Case presentation

A 50-year-old woman with a history of bronchiectasis, but no cardiovascular risk factors or toxic habits, presented with a nine-month history of progressively worsening dyspnoea and chronic cough. On admission, she was tachypnoeic and hypotensive, with jugular venous distension, marked peripheral oedema, and peripheral cyanosis. Cardiac auscultation revealed a grade III/VI systolic murmur along the left sternal border, consistent with tricuspid regurgitation, and a harsh systolic murmur over the pulmonary area radiating to the right hemithorax. Pulmonary auscultation was unremarkable, and abdominal examination showed no hepatosplenomegaly.

Chest radiography and routine laboratory investigations revealed a D-dimer level of 0.5 μg/mL (reference range 0.1–0.6 μg/mL). Cardiac troponin T was mildly elevated at 18.8 ng/L. Transthoracic echocardiography demonstrated marked right-sided chamber dilatation and a heterogeneous mass attached to the pulmonary valve and extending into the main pulmonary artery trunk (*Figure 1*). Bronchoscopy was considered as part of the diagnostic work-up but was cancelled because of active airway bleeding.

**Figure 1:**
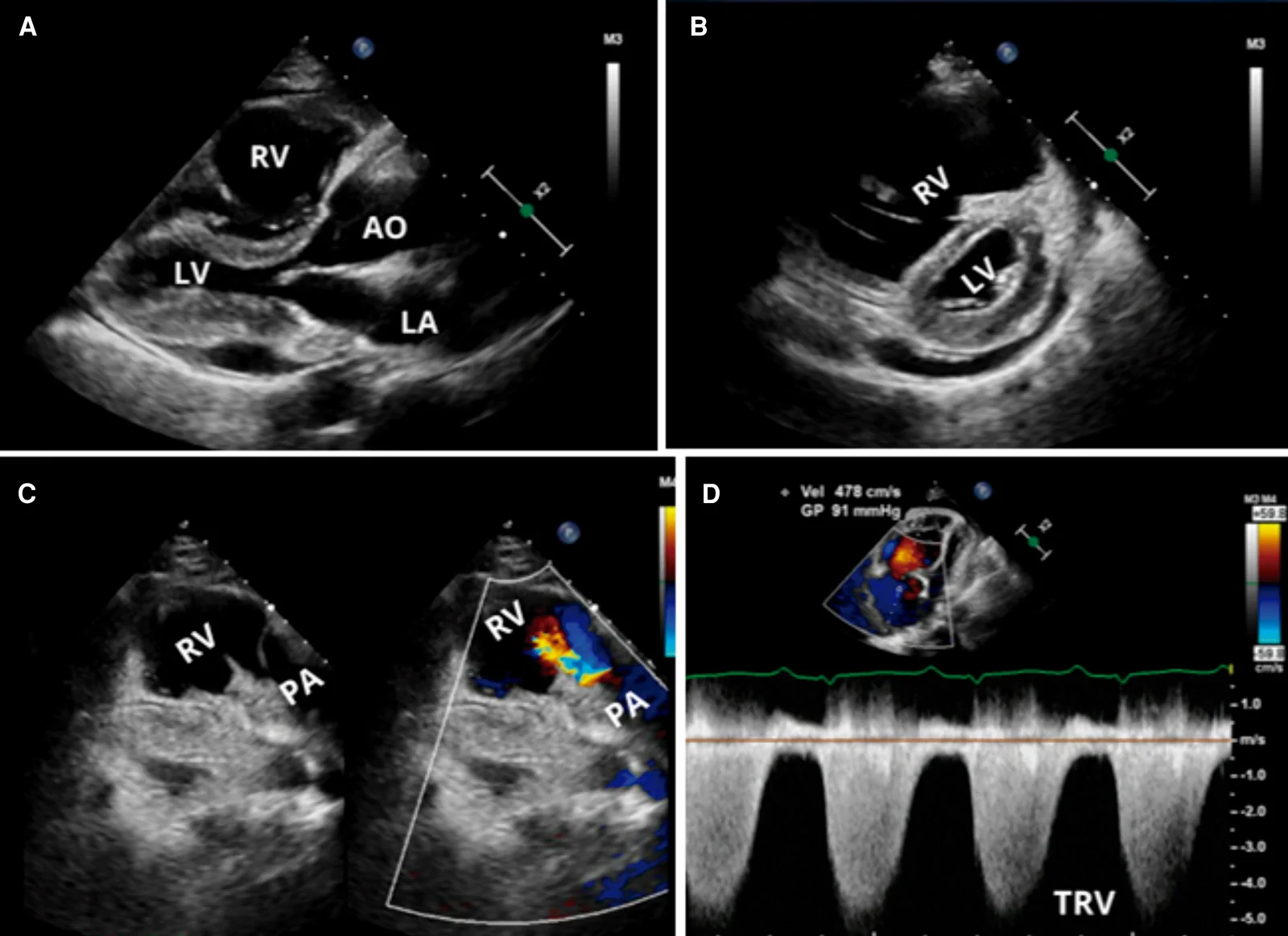
Transthoracic echocardiography. (*A*) Right ventricular dilatation with interventricular septal flattening. An apparent hyperechoic image adjacent to the proximal ascending aorta was considered inconclusive on transthoracic echocardiography and was not confirmed by computed tomography pulmonary angiography. (*B*) Parasternal short-axis view showing right ventricular enlargement with interventricular septal flattening and a small laminar pericardial effusion. (*C*) Masses visualized within the pulmonary artery trunk, with contiguous extension from the pulmonary valve into the main pulmonary artery. (*D*) Tricuspid regurgitation velocity of 4.8 m/s with an estimated right ventricular systolic pressure of 112 mmHg, indicating severe pulmonary hypertension.

Given the haemodynamic compromise and evidence of right ventricular overload, pulmonary embolism was initially suspected. Computed tomography pulmonary angiography (CTPA) revealed extensive intra- and extraluminal involvement of the pulmonary artery, with features suggestive of an underlying malignant process (*Figure 2*). However, pulmonary embolism with possible superimposed thrombus could not be excluded, and therapeutic anticoagulation was initiated in accordance with standard clinical practice. During hospitalization, the patient experienced two episodes of massive haemoptysis requiring admission to the intensive care unit. Management consisted of airway protection, supplemental oxygen therapy, intravenous crystalloid resuscitation, administration of tranexamic acid, and temporary withdrawal of anticoagulation because of active bleeding. Following clinical stabilization, anticoagulation was cautiously resumed. Bilateral lower extremity venous Doppler ultrasonography, performed as part of the diagnostic work-up for suspected pulmonary embolism, showed no evidence of deep vein thrombosis. An extensive rheumatological work-up, including testing for antiphospholipid syndrome, vasculitis, and systemic lupus erythematosus, was negative.

**Figure 2:**
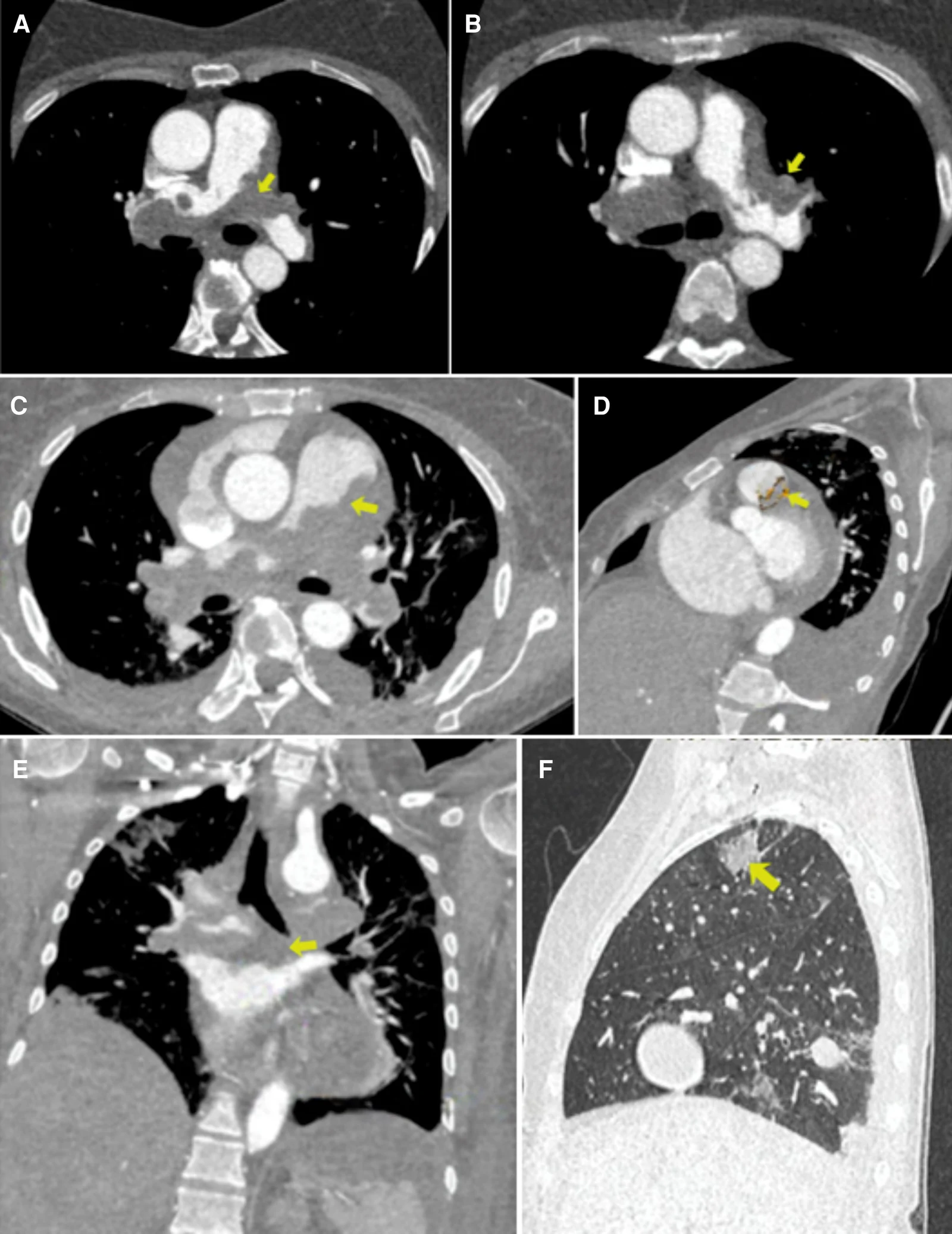
Follow-up chest CT pulmonary angiography. (*A*) Infiltrative mass involving the main pulmonary artery with luminal expansion. (*B*, *C*) Extension into both pulmonary artery branches. (*D*) Involvement of ∼20% of the pulmonary valve circumference. (*E*) Infiltration of the mediastinum and roof of the left atrium. (*F*) Multiple newly developed bilateral pulmonary nodules (lung window), consistent with metastatic spread.

Repeat transthoracic echocardiography demonstrated severe right ventricular dysfunction, a tricuspid regurgitation velocity (TRV) of 4.8 m/s, and an estimated right ventricular systolic pressure (RVSP) of 112 mmHg, consistent with severe pulmonary hypertension. Multiple irregular isoechoic masses were visualized within the main pulmonary artery, extending into both the right and left pulmonary arteries immediately distal to the pulmonary valve.

Subsequent CTPA showed marked disease progression, with an infiltrative mass extending from the pulmonary valve sinuses into the second-order pulmonary branches, causing up to 75% stenosis of the distal pulmonary trunk and sub-occlusive involvement of both pulmonary arteries. The lesion also extended into the adjacent mediastinum and infiltrated the roof of the left atrium. Multiple newly developed pulmonary nodules were identified (*Figure 2*).

The combination of rapid radiological progression, lack of response to anticoagulation, and worsening right ventricular failure prompted reconsideration of the initial diagnosis and referral to a tertiary centre for further evaluation, including consideration of surgical thromboendarterectomy.

During hospitalization, a firm palpable mass was detected in the left breast. Breast ultrasound classified the lesion as BI-RADS 5. Following multidisciplinary discussion, biopsy of the breast lesion was selected as the safest diagnostic approach. Histopathological analysis revealed a high-grade spindle-cell sarcoma with vascular differentiation. Immunohistochemistry showed strong positivity for CD31, ERG, and MDM2, while CD34, factor VIII, cytokeratins, and hormone receptors were negative, excluding primary breast carcinoma and supporting the diagnosis of metastatic vascular sarcoma of non-mammary origin (*Figure 3*). Surgical resection was not pursued because of poor functional status (ECOG performance status 4), extensive local invasion precluding complete resection, and evidence of metastatic disease.

**Figure 3:**
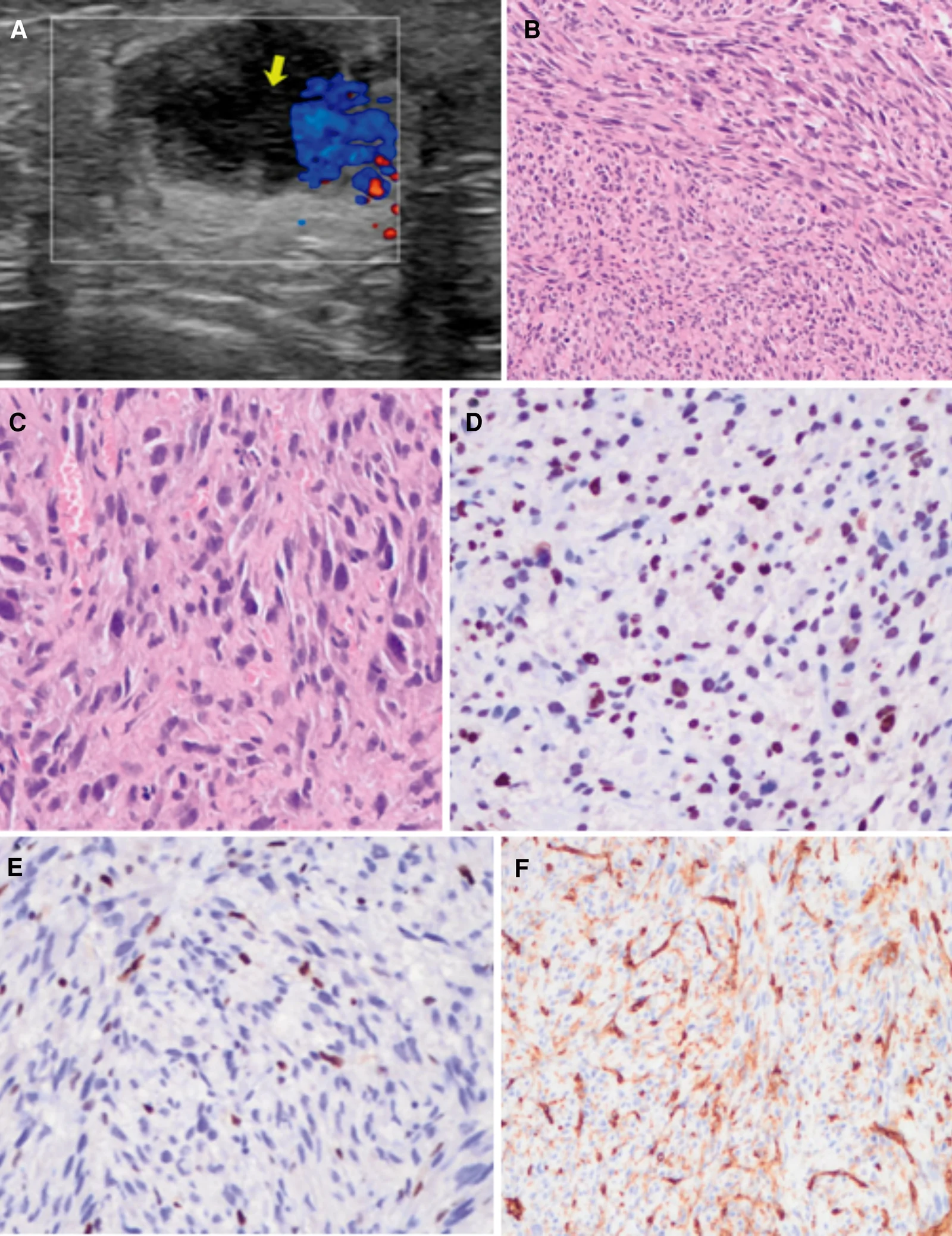
Histopathology of the breast lesion. (*A*) Breast Doppler ultrasound demonstrating a hypervascular solid lesion. (*B*) Proliferation of pleomorphic spindle cells with atypical mitoses and hyperchromatic nuclei (haematoxylin and eosin, ×100). (*C*) Pseudovascular spaces containing erythrocytes (haematoxylin and eosin, ×400). (*D*) MDM2 immunopositivity. (*E*) Nuclear ERG positivity. (*F*) CD31 positivity, confirming vascular differentiation.

## Discussion

Pulmonary artery sarcomas are broadly classified into luminal and intramural subtypes. The luminal form, most commonly represented by intimal sarcoma, is characterized by an undifferentiated spindle-cell phenotype and accounts for the majority of cases, whereas intramural sarcomas are typically leiomyosarcomas.^2^ Intimal sarcomas arise from the intimal layer of the pulmonary artery and predominantly grow intraluminally, causing progressive vascular obstruction. Less frequently, they may extend proximally to involve the pulmonary valve or, in advanced stages, the right ventricle.^7^

Clinical presentation is heterogeneous and often non-specific, ranging from chest pain and haemoptysis to progressive dyspnoea, syncope, and constitutional symptoms such as weight loss and anaemia.^8^ Local pulmonary involvement has been reported in ∼50% of patients, and distant metastases may occur in organs including the liver, pancreas, small bowel, and brain.^9^ Because these manifestations frequently overlap with those of pulmonary embolism, misdiagnosis is common and contributes substantially to diagnostic delay.

Contrast-enhanced CTPA is the most widely used initial imaging modality in this setting. Radiological features that should raise suspicion of a malignant intravascular process rather than thromboembolic disease include a mass that expands rather than occludes the vessel lumen, extraluminal extension, and heterogeneous contrast enhancement.^6,7^

In the present case, the diagnostic challenge was compounded by a clinical phenotype highly suggestive of pulmonary embolism, leading to anticoagulation in accordance with standard practice. However, several discordant findings—including a persistently normal D-dimer level, lack of clinical or radiological response to anticoagulation, and rapid progression of the pulmonary artery mass with newly developed pulmonary nodules—prompted reconsideration of the initial diagnosis. Histopathological confirmation was ultimately achieved by biopsy of a breast lesion, representing an unusual diagnostic pathway. While previous reports have described diagnosis through pulmonary endarterectomy specimens or endovascular biopsy, confirmation by biopsy of an extrathoracic metastasis has rarely been reported,^10^ highlighting the exceptional nature of this presentation.

Prognosis in pulmonary artery intimal sarcoma remains poor and is largely determined by tumour extent and resectability. Median survival is ∼1.5 months without intervention and may extend to 10–12 months in selected patients undergoing complete surgical resection.^4^ The role of adjuvant chemotherapy or radiotherapy remains uncertain, and there is no consensus regarding the optimal use or duration of anticoagulation in this setting.^3,4^

Catheter-directed therapies and mechanical thrombectomy are increasingly used in acute pulmonary embolism. However, these interventions are designed to remove thrombotic material. In pulmonary artery intimal sarcoma, obstruction is caused by malignant tumour rather than thrombus; therefore, thrombectomy may be ineffective and potentially harmful. Recognition of diagnostic discordance is essential to avoid inappropriate invasive intervention.^5,11^

This case illustrates the diagnostic challenge posed by pulmonary artery intimal sarcoma and emphasizes the importance of maintaining a broad differential diagnosis in patients with suspected pulmonary embolism who present with discordant clinical, laboratory, or imaging findings. Early recognition and referral to specialized centres remain essential to improve diagnostic accuracy and optimize patient management.

## Lead author biography


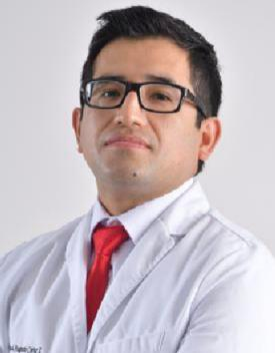


Maicol Cortez Sandoval is a cardiologist at the National Hospital Edgardo Rebagliati Martins in Lima, Perú. He holds a Master’s in Clinical Cardiology from the Universidad Católica de Murcia and a Master’s in Cardiac Arrhythmology from Universidad Rey Juan Carlos. Additionally, he has specialized training in heart failure, coronary artery disease, and cardiac magnetic resonance imaging. Dr Cortez is dedicated to advancing cardiovascular care and research, with a particular focus on arrhythmias.

## Data Availability

The data underlying this article are included within the article. Additional data are available from the corresponding author upon reasonable request.

## Notes

**Consent:** The authors confirm that written consent has been obtained from the patient for the submission and publication of this case report, including images and associated clinical information, in accordance with the Committee on Publication Ethics guidance.
